# Different gastric microbiota compositions in two human populations with high and low gastric cancer risk in Colombia

**DOI:** 10.1038/srep18594

**Published:** 2016-01-05

**Authors:** Ines Yang, Sabrina Woltemate, M. Blanca Piazuelo, Luis E. Bravo, Maria Clara Yepez, Judith Romero-Gallo, Alberto G. Delgado, Keith T. Wilson, Richard M. Peek, Pelayo Correa, Christine Josenhans, James G. Fox, Sebastian Suerbaum

**Affiliations:** 1 Institute of Medical Microbiology and Hospital Epidemiology, Hannover Medical School, Carl-Neuberg-Str. 1, 30625 Hannover, Germany; 2 German Center for Infection Research, Hannover-Braunschweig Site, Carl-Neuberg-Str. 1, 30625 Hannover; 3 Division of Gastroenterology, Department of Medicine, Vanderbilt University, Nashville, TN, USA; 4 Department of Pathology, Universidad del Valle, Cali, Colombia; 5 Centro de Estudios en Salud, Universidad de Nariño, Pasto, Colombia; 6 Department of Cancer Biology, Vanderbilt University, Nashville, TN, USA; 7 Veterans Affairs Tennessee Valley Healthcare System, Nashville, TN, USA; 8 Division of Comparative Medicine, Massachusetts Institute of Technology, Cambridge, MA, USA

## Abstract

Inhabitants of Túquerres in the Colombian Andes have a 25-fold higher risk of gastric cancer than inhabitants of the coastal town Tumaco, despite similar *H. pylori* prevalences. The gastric microbiota was recently shown in animal models to accelerate the development of *H. pylori*-induced precancerous lesions. 20 individuals from each town, matched for age and sex, were selected, and gastric microbiota analyses were performed by deep sequencing of amplified 16S rDNA. In parallel, analyses of *H. pylori* status, carriage of the *cag* pathogenicity island and assignment of *H. pylori* to phylogeographic groups were performed to test for correlations between *H. pylori* strain properties and microbiota composition. The gastric microbiota composition was highly variable between individuals, but showed a significant correlation with the town of origin. Multiple OTUs were detected exclusively in either Tumaco or Túquerres. Two operational taxonomic units (OTUs), *Leptotrichia wadei* and a *Veillonella sp.*, were significantly more abundant in Túquerres, and 16 OTUs, including a *Staphylococcus sp.* were significantly more abundant in Tumaco. There was no significant correlation of *H. pylori* phylogeographic population or carriage of the *cag*PAI with microbiota composition. From these data, testable hypotheses can be generated and examined in suitable animal models and prospective clinical trials.

With an estimated 1 million new cases per year, and 720,000 deaths, gastric cancer is a leading cause of morbidity worldwide, and the third most common cause of cancer-related death^1^. *Helicobacter pylori* infection is the best-studied risk factor for gastric cancer, and 89% of non-cardia gastric cancer cases are estimated to be attributable to *H. pylori* infection^1^. However, cancer risk can vary dramatically between populations with relatively similar prevalence of *H. pylori* infection. The Colombian department of Nariño in the southwest of the country includes both coastal regions, where stomach cancer is comparatively rare (6 cases per 100 000 inhabitants in 1976), and high-altitude parts of the Andes, where gastric cancer rates are strikingly high (150 cases/100,000 inhabitants in 1976)^2^. The contrasting rates of gastric cancer are associated with very similar rates of *H. pylori* infection between regions, with 95% of inhabitants testing seropositive for the pathogen both in coastal and in high-altitude regions^3^.

Despite four decades of research, the reasons for this “altitude enigma” remain incompletely understood. It has been suggested that altitude is a surrogate for multifactorial influences of host and bacterial genotypes, as well as dietary and environmental variables which may all contribute to the effect^2^,^4^,^5^,^6^. We and others have demonstrated the high incidence of helminthiasis in select Colombian populations, particularly children^3^. Our studies^3^ suggest that the immune response to *H. pylori* infection in the low-risk coastal population is predominantly type Th2 and that it may be related to intestinal helminthiasis. This observation is consistent with our report that intestinal helminthiasis reduced gastric atrophy, a premalignant lesion, in the C57BL/6 mouse model of *Helicobacter* gastritis^7^. Our recent studies in insulin-gastrin (INS-GAS) mice demonstrating a *H. pylori-* associated attenuation of premalignant lesions in these mice coinfected with the nematode *Heligmosomoides polygyrus* also support this hypothesis^8^.

Recently, Kodaman *et al.*^9^ demonstrated that cancer risk was highest in individuals whose host and bacterial ancestries were mismatched, possibly disrupting a balance generated by thousands of years of host-microbe coevolution. Cancer risk was particularly high when individuals of Amerindian descent carried *H. pylori* with largely African ancestry, while individuals with matching host ancestry and *H. pylori* strain had a lower risk.

After a period where *H. pylori* was thought to be the only physiologically relevant bacterial colonizer of the human gastric mucosa, several studies provided evidence that bacteria other than *H. pylori* can regularly be detected in gastric biopsies, although the ecological role of these bacteria remains unclear. However, several lines of evidence point at a potential role of the microbiota in gastrointestinal carcinogenesis. We have recently shown in a transgenic mouse model of gastric carcinogenesis, the INS-GAS mouse, that the presence of a gastrointestinal microbiota strongly accelerated the induction of gastric preneoplasic lesions by *Helicobacter pylori*^10^. This study was further supported in the INS-GAS mono-associated *H. pylori* model where the addition of a select intestinal microflora accelerated gastric cancer^11^. These studies raise the possibility that the non-*H. pylori* gastric microbiota contributes to gastric carcinogenesis, and that components of the gastric microbiota may play a role in causation and/or serve as biomarkers of gastric cancer risk.

In this study, we have analyzed the composition of the gastric microbiota of individuals from the Colombian high-risk and low-risk areas of Túquerres and Tumaco, respectively. The data show significant differences between towns, and permitted us to identify bacterial species that only occurred in either region, generating testable hypotheses for future clinical and experimental studies.

## Results

### Gastric microbiota composition in individuals from Tumaco and Túquerres (Colombia)

Antral gastric biopsies from two groups of 20 individuals each from two cities in Colombia, Tumaco, a coastal town with low gastric cancer risk, or Túquerres, a town in the Andes mountains with high gastric cancer risk, were subjected to gastric microbiota analysis. Individuals were matched by age and sex (Table 1), and intentionally selected with similar assignment of gastric disease.

DNA was purified from the biopsies using a DNA extraction protocol optimized for efficient lysis of diverse bacterial taxa, a fragment of the conserved 16S rDNA gene was amplified with a set of broad range primers recognizing highly conserved sequence motifs, and the amplicons were then sequenced with high coverage using Roche 454 FLX + technology.

The full dataset (before subsampling) consisted of a total of 647,914 sequences, with 6960 to 32,147 sequences per sample (Supplementary Dataset S1). 555,430 of these sequences could be identified to species level (85.7%), resulting in a total of 187 species and 575 97% identity clusters of sequences not identified to the species level. For the purpose of this study, both identified species and 97% identity clusters were considered as operative taxonomic units (OTUs).

Where not noted otherwise, analyses were based on a rarefied version of this dataset, which consisted of 6960 sequences per sample (total number of sequences, 278,400; for rarefaction curves see Supplementary Fig. 1). Within this subsampled dataset, 229,384 sequences (82%) were identified to species level; it contained 125 identifiable species and 299 other OTUs (Supplementary Dataset S2). Individual samples contained 4 to 199 OTUs (Supplementary Dataset S2), which included 1 to 6952 sequences identified as members of the *Helicobacteraceae* (Supplementary Dataset S2, Supplementary Table S1). *Helicobacteraceae* sequences not identified to species were treated as potential members of the species *H. pylori*.

**Figure 1:**
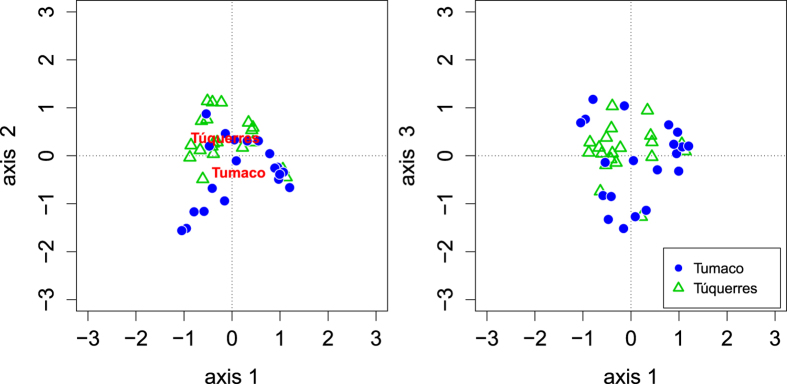
PCoA analysis of the subsampled microbiota data, based on UniFrac distances, with significantly correlated sample characteristics fitted to the graph (p <  = 0.05; for p values see table 2).

We first asked whether the microbiota composition differed between the two towns, and whether the microbiota composition was correlated with patient characteristics such as histological diagnosis. To accomplish this, we used Principal Coordinates Analysis (PCoA) of the microbiota data and fitted patient characteristics and town of origin onto the resulting graphs. This analysis was based on unweighted UniFrac distances (Fig. 1), which incorporate the phylogenetic relatedness of the OTUs in the dataset when comparing the similarity between samples. The town of origin was found to be significantly correlated with the pattern of microbiota composition (envfit factor fitting, confirmed by Analysis of Molecular Variance AMOVA based on Jaccard index dissimilarities, p = 0.004, n = 40). No significant correlations were obtained between the microbiota composition and histological score, diagnosis, patient sex or patient age, respectively (Table 2).

### Distribution patterns of individual microbiota components between towns

**Figure 2:**
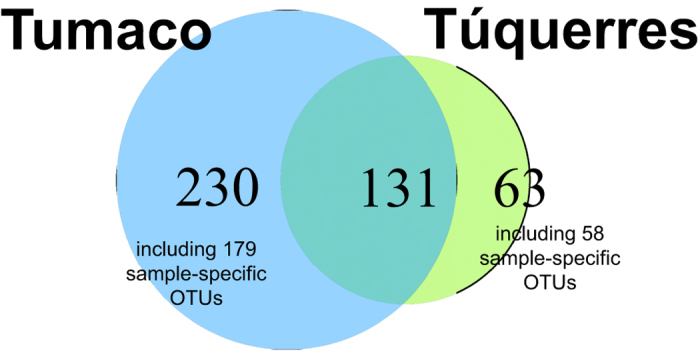
Venn diagram visualizing the number of town-specific and shared OTUs. Numbers of sample-specific OTUs indicated.

We next analysed whether the individual bacterial OTUs occurred more abundantly in individuals from either Tumaco or Túquerres. The presence and abundance of individual OTUs was highly variable between individuals (Fig. 2, Supplementary Dataset S2). Nevertheless, Metastats-based analysis of differential abundances^12^ identified 2 OTUs that were significantly more abundant in Túquerres and 16 OTUs significantly more abundant in Tumaco individuals (false discovery rate q < 0.05, 20 samples per town, sample-specific OTUs excluded; Table 3). The OTUs significantly more abundant in Túquerres were identified as *Leptotrichia wadei* and as a member of the genus *Veillonella*, respectively. While the OTUs that were significantly more abundant in the Túquerres group could also be detected in individual samples from Tumaco, the OTUs significantly more abundant in the Tumaco patient group were not identified in the samples from Túquerres (in the subsampled dataset). The Tumaco-specific bacteria included an OTU identified as a member of the genus *Staphylococcus* which was found in 35% of the Tumaco samples (7 of 20 samples), the species *Neisseria flavescens* (4 samples), a member of the family *Porphyromonadaceae* (4 samples), an OTU of the genus *Flavobacterium* (4 samples), and an OTU belonging to the candidate division TM7 (3 samples).

Based on the rooted phylogenetic tree that had been constructed for the calculation of UniFrac distances (Supplementary Fig. S2), we next tested whether additional phylogenetic groups above the OTU level were differently abundant between the Tumaco and Túquerres individuals. We used the phylogenetic tree to calculate the branch length difference (cophenetic distance) between the individual OTUs (using cophenetic.phylo of the R package phyloseq). We examined clades at the distance levels commonly used as proxy for the difference between species (cophenetic distance 0.03), genera (cophenetic distance 0.05) and families (cophenetic distance 0.1). The dataset’s OTUs were merged at these three distance levels (using tip_glom of the phyloseq package), and the resulting merged OTUs were examined using Metastats. While most clades identified as being significantly different in abundance between Tumaco and Túquerres contained individual OTUs that had already been identified as being different in abundance in the first analysis, some additional clades were identified as differently abundant between towns: At a cophenetic distance of 0.03 (“approximate species level”), 4 additional multi-OTU-clades were found to be significantly more abundant in Tumaco. This included two Tumaco-specific clades of OTUs classified as *Actinomyce*s spp., one clade consisting of *Streptomyces* spp. and another clade of OTUs identified as *Catonella* spp. (Supplementary Dataset S3). No additional significantly different clade was identified at a cophenetic distance of 0.05 (“approximate genus level”) (Supplementary Dataset S4). At a cophenetic distance of 0.1 (“approximate family level”), three additional clades were identified as significantly more abundant in Tumaco. These were identified as a clade of *Flavobacteriaceae*, members of the genus *Prevotella*, and members of the genus *Mycoplasma*, respectively (Supplementary Dataset S5).

We further tested for direct associations of patient characteristics with individual non-*H. pylori* OTUs (Supplementary Dataset S6). This analysis was based on three versions of the OTU dataset: an abundance-based and a binary version of the subsampled dataset as well as an abundance-based non-subsampled version of the full OTU dataset. From all three versions, all *Helicobacteraceae* OTUs were removed. While no OTU was correlated with the histopathology score, a number of OTUs were correlated with the diagnosis of MAG-IM (categorical factor regression; false discovery rate ≤0.05). Most of these were identified as members of oral and respiratory tract groups, including several OTUs classified as *Actinomyces*, *Prevotella* and *Streptococcus*. Correlation analysis based on Spearman’s rank correlation (false discovery rate ≤0.05) identified one *Streptococcus* OTU positively correlated with the number of *Helicobacteraceae* sequences in the subsampled abundance-based dataset. The species *Actinomyces odontolyticus*, which was also one of the OTUs associated with MAG-IM, was identified as positively correlated with patient age in both the abundance-based and the binary subsampled dataset; it occurred both in the Tumaco and the Túquerres groups. In the non-subsampled dataset, a *Pseudomonas* OTU, an OTU identified as a member of *Xanthomonadaceae* and an OTU of the genus *Prevotella* were found to be correlated with age (Supplementary Dataset S6).

### *H. pylori* and the gastric microbiota composition

**Figure 3:**
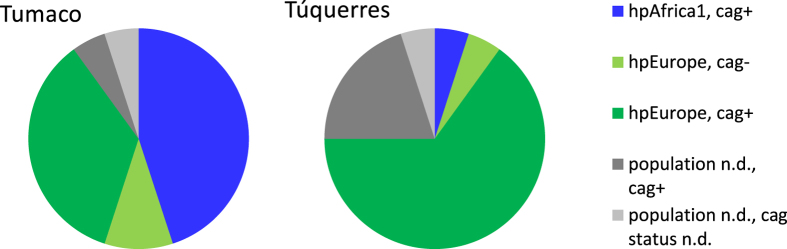
Overview over *H. pylori* population assignment and sample *cag*PAI status. N = 20 for each town.

**Figure 4:**
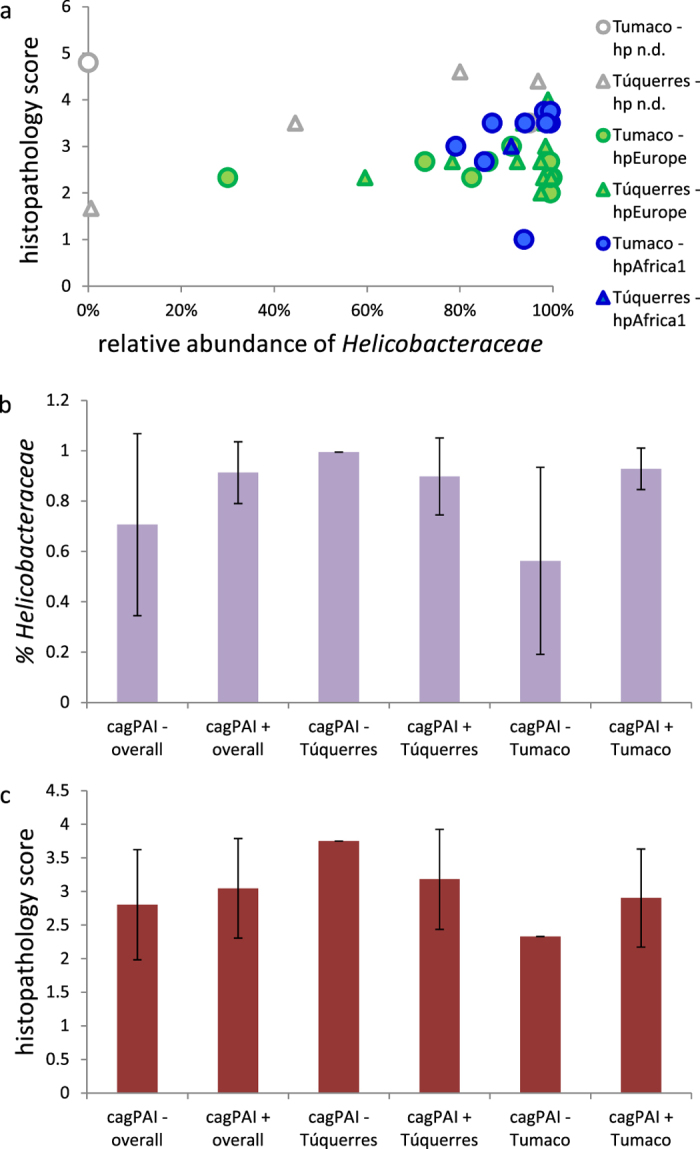
Histopathology score and relative abundance of *Helicobacteraceae* in the full dataset in relation to *H. pylori* population and *cag*PAI status. (**a**), histopathology score *vs.* abundance of *Helicobacteraceae*, plotted by *H. pylori* population. (**b**), abundance of *Helicobacteraceae* by *cag*PAI status and town. (**c**), histopathology score by *cag*PAI status and town. For p values see Supplementary Dataset S7.

The presence of *H. pylori* infection alters gastric physiology and is thus likely to affect the gastric microbiota. While all but one individual included in this study were initially tested as *H. pylori*-positive by histology, and while *H. pylori* sequences could be identified from all samples, infecting *H. pylori* strains were diverse. *H. pylori* can be subdivided into phylogeographic populations with distinct geographic distribution and differential carriage of virulence factors. Specific combinations of host ancestry and *H. pylori* populations were recently shown to be associated with more severe gastric lesions in individuals from a Colombian patient cohort similar to our cohort^9^. In order to evaluate a potential influence of *H. pylori* phylogeography on the gastric microbiota composition, we performed multilocus sequence analysis (MLSA) for *H. pylori* strains isolated from biopsy samples from the same individuals for whom the microbiota analysis had been performed. *H. pylori* isolates were available for 15 samples from Túquerres and 18 samples from Tumaco. One *H. pylori* isolate from Túquerres and 9 isolates from Tumaco were assigned to the *H. pylori* population hpAfrica1 (Túquerres isolate: subpopulation hspSAfrica; the 9 hpAfrica1 Tumaco isolates: subpopulation hspWAfrica); the remaining isolates were all identified as hpEurope (Fig. 3, Supplementary Table S2). In our dataset, a difference in gastric lesions associated with hpAfrica1 and hpEurope strains could only be detected for Tumaco samples, in which hpAfrica1 strains were associated with significantly higher histopathology scores (3.13 ± 0.87 *vs.* 2.48 ± 0.30, p = 0.012, Wilcoxon rank sum test, see Supplementary Dataset S7, Fig. 4a). When comparing samples associated with hpEurope strains between towns, histopathology scores from Túquerres were significantly higher than those from Tumaco (2.98 ± 0.63 vs. 2.48 ± 0.30, mean ± standard deviation; p = 0.019, Student’s t-test with Welch’s correction, Supplementary Dataset S7). This difference was not detected between the complete sample sets of the two towns. Modern *H. pylori* strains are mosaics that have arisen by recombination between members of six known ancestral *H. pylori* populations. The composition of the *H. pylori* isolates from the six know ancestral *H. pylori* populations was investigated with an additional STRUCTURE analysis assuming recombination of the 6 known ancestral *H. pylori* populations according to the linkage model. Among the samples from Túquerres, the maximum proportion of Ancestral Africa 1 (AA1) ancestry (32.9%) was found in the strain identified as hspSAfrica; four other Túquerres strains had more than 20% AA1 ancestry (22.5–24.1%). Among the Tumaco isolates, 8 of the 18 isolates were predominantly (>50%) of AA1 ancestry, and a total of 12 samples were identified as more than 20% AA1 (Supplementary Table S2, Supplementary Table S3). A significant correlation between histopathology scores and proportion of AA1 ancestry was detected for Tumaco samples (rho = 0.53, p = 0.022), but not for the samples from Túquerres (Supplementary Dataset S7).

We next tested for correlations between population and ancestry of the infecting *H. pylori* strains and the respective stomach microbiota (factor fitting on the PCoA ordination). Neither the *H. pylori* population nor the proportion of AA1 ancestry was found to be significantly correlated with the microbiota composition (Table 2). In addition, no individual OTU was found to be correlated with *H. pylori* population or the proportion of AA1 ancestry.

Independently of the *H. pylori* population, presence of the *cag* pathogenicity island (*cag*PAI) is an important determinant of *H. pylori* virulence. Using several PCR assays on both biopsy samples and individual *H. pylori* strains for each patient, we could assign a *cag*PAI status to 19 samples for each location (Fig. 3, Supplementary Table S2). While most samples (35) tested positive, one sample from Túquerres and two samples from Tumaco were c*ag*PAI*-*negative. All three *cag*PAI-negative strains were assigned to the *H. pylori* population hpEurope (Fig. 3, Supplementary Table S2). Both in the full dataset and in the Tumaco samples, the *cag*PAI-negative status was associated with lower abundances of *Helicobacteraceae* sequences and histopathology scores. Due to the low number of *cag*PAI-negative samples and non-normality of the data, significance of these differences could not be assessed (Fig. 4b,c; Supplementary Dataset S7). While factor fitting on the PCoA analysis identified no correlation of *cag*PAI status with the overall microbiota composition (Table 2), regression analysis identified 9 OTUs of the non-subsampled abundance-based dataset as correlated with a negative *cag*PAI status (Supplementary Dataset S6).

## Discussion

Human populations with similarly high *H. pylori* prevalence can display strong differences of gastric cancer risk. This has motivated intensive studies of both, differences of carcinogenic potential between *H. pylori* strains and diverse co-carcinogenic factors including host susceptibility and environmental conditions. In this study, we have analyzed the composition of the gastric microbiota of two human populations in Colombia with starkly different gastric cancer risks, with the aim to identify microbiota components that might be involved in the development of gastric cancer initiated by chronic *H. pylori* infection, or serve as biomarkers of gastric cancer risk.

Recent studies in animal models have provided evidence of a potential role of the non-*H. pylori* microbiota in *H. pylori*-induced gastric carcinogenesis^10^,^11^,^13^. The gastric microbiota was shown to accelerate and enhance the development of preneoplastic lesions and adenocarcinoma in the transgenic INS-GAS mouse model^10^,^11^. The complete microbiota or individual microbiota components were reported to add additional noxious effects by formation of carcinogenic nitrosamines in hypochlorhydric stomachs^14^, or beneficial effects, by reducing the production of pro-inflammatory cytokines^15^,^16^,^17^,^18^, increasing gastric ulcer healing^13^,^19^, or inhibiting *H. pylori* growth and colonization^15^,^20^. The stomach microbiota in turn can be influenced by *H. pylori* infection, at least in gerbils and mice^10^,^21^,^22^. Also, the presence of intestinal helminths in the *H. pylori* INS-GAS mouse model ameliorated gastric atrophy and dysplasia which are important precursor lesions to gastrointestinal intraepithelial neoplasia (GIN). Helminth co-infection resulted in increased Foxp3 cells in the corpus and inhibited gastric colonization with enteric bacteria^8^.

Our data clearly show that the gastric microbiota composition differed between the two towns. Most of the OTUs that we identified as significantly more abundant in either high-risk Túquerres or low-risk Tumaco (Table 3) were classified as taxa previously identified in “healthy” human microbiomes, and in stomach samples^23^,^24^,^25^,^26^,^27^,^28^,^29^,^30^,^31^,^32^,^33^. Nevertheless, one of the OTUs significantly more abundant in Túquerres, the fusobacterium *Leptorichia wadei*, can be associated with necrotizing enterocolitis and bacteremia in chemotherapy patients^34^. Among the OTUs that might be associated with the lower cancer risk of the Tumaco inhabitants, *Staphylococcus* OTU 566 occurred in 9 Tumaco samples and in one of the samples from Túquerres (non-subsampled full dataset). The genus *Staphylococcus* is part of the human normal microbiota, and several species of this genus were previously reported in stomach samples^30^,^31^,^33^. Another Tumaco-associated OTU is the species *Streptococcus oralis*, an oral cavity commensal^35^,^36^, which was found to be significantly less abundant in endodontic infections than in other parts of oral cavity^37^. These two OTUs might warrant further investigation regarding a possible involvement in protection against inflammatory processes and cancer development.

Further interesting OTUs include the Tumaco-specific OTU 486, which was classified as belonging to the genus *Haematobacter*, a recently described genus that is most often isolated from human blood, although the type strain was isolated from the nose of a patient with aspiration pneumonia^38^. Among the few available studies on this genus is a case report of endocarditis possibly caused by a *Haematobacter*-like organism^39^. OTU 430, classified as *Rothia* sp., and *Capnocytophaga gingivalis* were both associated with the town of Tumaco, and OTU 430 was additionally correlated with a diagnosis of MAG-IM. Both the genus *Rothia* and the species *Capnocytophaga gingivalis* are otherwise associated with healthy oral surfaces^29^,^35^,^36^,^37^,^40^, and the genus *Rothia* is regularly found in stomach samples^24^,^28^,^30^,^31^,^33^. As both these taxa are not normally linked to exacerbation of inflammatory or carcinogenic processes, both probably represent mainly swallowed organisms that can survive longer or bloom in more physiologically compromised (and probably less acidic) stomachs.

Similarly to the town-associated OTUs, most of the OTUs identified as correlated with a diagnosis of MAG-IM (Supplementary Dataset S6) were identified as taxa previously identified from stomach samples^23^,^24^,^25^,^26^,^27^,^28^,^29^,^30^,^31^,^32^,^33^,^41^. All but one belong to taxa that are part of the normal flora of the human mouth^29^,^35^,^36^,^37^,^40^,^42^,^43^,^44^, and many are also found in intestinal samples^29^; the exception to this is OTU 319, a member of the genus *Pelomonas* that is otherwise associated with water samples^45^,^46^. In spite of their occurrence in healthy microbiome samples, several MAG-IM-associated OTUs are known to be linked to inflammatory pathologies, albeit in the mouth^37^,^47^. This includes two representatives (OTU 492 and *P. oris*) of the genus *Prevotella*, which contains several periodontal pathogens. The genus *Granulicatella*, which is represented by *G. adiacens* and OTU 336, has been linked to root canal infections^37^; *Mogibacterium timidum* and *Anaeroglobus geminatus* are periodontal pathogens^47^, and the genus *Eubacterium* (OTU 461) contains a newly identified oral pathogen, the species *Eubacterium saphenum*. Additionally, four members of the genus *Streptococcus* were correlated with MAG-IM: OTU 327 and the species *S. infantis, S. salivarius* and *S. sanguinis*. Finding such a high number of *Streptococcus* OTUs to be associated with pathological lesions was surprising, but is probably due to the high abundance and diversity of this genus in our samples: the subsampled dataset contains 61 OTUs classified as *Streptococcus* spp. (Supplementary Dataset S2). Conversely, although streptococci are members of the healthy human oral microbiota that are regularly found in stomach samples^24^,^28^,^30^,^31^,^33^, an increased abundance of this genus in antral gastritis and in peptic ulcer disease has been reported^28^,^33^. Interestingly, many of the *Streptococcus* species are urease positive, as are several *Staphylococcus* species, and their presence in the stomach, in part, may be attributed to the enzyme which provides a protective advantage for survival in an acidic environment. In our dataset, the only non-*Helicobacteraceae* OTU that significantly increased in abundance with increasing dominance of *Helicobacteraceae* was also classified as *Streptococcus* sp. (OTU 568). An unidentified member of this genus was previously reported to be part of a probiotic mix that accelerated gastric ulcer healing in rats by inducing the expression of growth factors which enhance processes such as epithelial cell proliferation and angiogenesis^19^. On the other hand, the species *S. sinensis* was found to be more abundant in NAG than in MAG-IM and even less abundant in gastric cancer^48^.

The low pH of the gastric lumen, together with the activity of pepsins, is the major reason why the stomach is relatively poor in bacteria. Conditions affecting gastric acidity are likely to have a major influence on the gastric microbiota. Overgrowth of the stomach microbiota is a known complication of acid-suppressive therapy. As mentioned above, we thus cannot exclude that the increased occurrence of the MAG-IM-correlated OTUs might result from increased survival in MAG-IM damaged stomachs which have an elevated pH. Nevertheless, they represent candidates that might be of interest for targeted studies on the influence of individual taxa on the development of gastric pathology.

We note that the ecology of the gastric microbiota has been studied very little. Open questions include the stability of the gastric microbiota over time and the differentiation between passing bacteria and bacteria causing a stable colonization. A comparative analysis of saliva and mucosal gastric microbiota samples from individuals from a cohort with contrasting gastric cancer risks might help to resolve these questions.

The differences in stomach cancer risk in high vs. low altitude regions in Colombia have been linked to a variety of factors, including differences of the colonizing *H. pylori* strains. In order to evaluate whether the microbiota composition was independent of *H. pylori* type, or rather reflected differences of the colonizing *H. pylori* strain, we also characterized one *H. pylori* isolate per sample and tested DNA extracted from biopsies for selected genes of the *cag*PAI. In agreement with previous studies from the region, 50% of *H. pylori* strains isolated from low-risk Tumaco were assigned to the *H. pylori* biogeographic population hpAfrica1, while the remainder of the Tumaco strains and almost all strains from the high-risk region around Túquerres were hpEurope^49^. However, no significant correlations of the overall gastric microbiota composition with either *H. pylori* population type or carriage of the *cag*PAI were detected, indicating that the differences of gastric microbiota composition in our analysis were largely independent of the colonizing *H. pylori* strains. We note that *cag*PAI-deficient *H. pylori* were present in the respective individuals in lower abundance than *cag*PAI positive bacteria, although significance of this finding could not be assessed statistically. The *cag*PAI has been suggested to provide a fitness benefit to *H. pylori*, but different densities of *H. pylori* for *cag*PAI-positive vs. -negative bacteria had not been reported before, an observation that warrants further investigations.

A recent study has demonstrated enhanced virulence of *H. pylori* strains with higher proportion of Ancestral Africa 1 (AA1) ancestry in human hosts with predominantly Amerindian ancestry^9^. This is concordant with the observed difference between high- and low-risk regions, because the inhabitants of high-risk Andean regions are mainly of Amerindian and European ancestry, while the inhabitants of the coastal low-risk region are mainly of African ancestry with significant proportions of Amerindian and European ancestry. Although human genetic data are not available for the individuals of our cohort, based on the published data, we expected a correlation between biopsy histopathology score and the fraction of AA1 ancestry in the *H. pylori* strains for samples from Túquerres only. In our data however, such a correlation was only detected in Tumaco (Supplementary Dataset S7). This might be due to the relatively small number of Túquerres subjects harbouring *H. pylori* with increased AA1 ancestry within this study, which was designed to test differences of microbiota composition in subjects matched for age, sex, and pathology scores.

Our study shows differences of microbiota composition between the two populations. From these data, testable hypotheses can be generated that can be examined in suitable animal models (e.g. the INS-GAS model) where candidate strains can be examined for their accelerating or protective effect on the development of *H. pylori*-induced preneoplastic lesions. Such studies are now under way in our laboratories. Even if our current study does not permit us to infer causality between microbiota components and pathology, microbiota composition data may prove informative about environmental factors that contribute to gastric cancer risk. As one example, dietary differences between the regions include higher consumption of fresh vegetables, fruit and seafood in the low-risk regions and higher consumption of potatoes and fava beans in the high-risk region^4^, which may contribute to differences in both microbiota and stomach pathologies.

## Methods

### Study participants, samples and histopathology

Subjects between 40 and 60 years old with dyspeptic symptoms that warranted upper gastrointestinal tract endoscopy were recruited in Tumaco and Túquerres in 2010. Subjects that had received proton pump inhibitors, H2-receptor antagonists, or antimicrobials during the 30 day period previous to the endoscopic procedure were excluded from this study. Other exclusion criteria were major diseases or previous gastrectomy. Participation was voluntary and informed consent was obtained from all participants. The Ethics Committees of the participating hospitals in Nariño and the Universidad del Valle in Cali, Colombia and the Institutional Review Board of Vanderbilt University approved all study protocols, and all experiments were performed in accordance with the relevant guidelines and regulations. See Supplemental Methods for details.

### DNA techniques and *H. pylori* multilocus haplotype analysis

See Supplemental Methods for details

### Microbiota analysis and bioinformatics analysis

The microbiota composition was analysed as described in Yang *et al.*^50^, with slight modifications. 16S rDNA amplicon reads were submitted to the European Nucleotide Archive under study accession number PRJEB11763. See Supplemental Methods for details.

## Additional Information

**How to cite this article**: Yang, I. *et al.* Different gastric microbiota compositions in two human populations with high and low gastric cancer risk in Colombia. *Sci. Rep.* **6**, 18594; doi: 10.1038/srep18594 (2016).

## Supplementary Material

Supplementary Information

Supplementary Dataset 1

## Figures and Tables

**Table 1:** Samples used in this study, with characteristics of the corresponding study participants.

Sample ID	Sample pair	Sex of participant	Age of participant	Location	Histo-pathological diagnosis	Histo-pathological score	*H. pylori* status
MT5106	1	F	40	Tumaco	NAG	2.33	Positive
MT2108	1	F	41	Túquerres	MAG	3.5	Positive
MT5136	2	F	41	Tumaco	NAG	3	Positive
MT2118	2	F	41	Túquerres	NAG	2.67	Positive
MT5176	3	M	42	Tumaco	NAG	2.33	Positive
MT2120	3	M	41	Túquerres	NAG	3	Positive
MT5139	4	F	43	Tumaco	NAG	1	Positive
MT2131	4	F	41	Túquerres	MAG	3.5	Positive
MT5117	5	F	44	Tumaco	MAG	3.75	Positive
MT2133	5	F	42	Túquerres	MAG	3.75	Positive
MT5124	6	F	46	Tumaco	MAG	3.5	Positive
MT2124	6	F	43	Túquerres	NAG	2.67	Positive
MT5135	7	M	45	Tumaco	MAG	3.5	Positive
MT2130	7	M	46	Túquerres	NAG	2.67	Positive
MT5101	8	F	47	Tumaco	NAG	2.33	Positive
MT2102	8	F	44	Túquerres	MAG	3.5	Positive
MT5105	9	F	47	Tumaco	MAG	3.5	Positive
MT2122	9	F	45	Túquerres	NAG	3	Positive
MT5119	10	F	48	Tumaco	MAG	3.5	Positive
MT2115	10	F	50	Túquerres	NAG	2.33	Positive
MT5120	11	F	48	Tumaco	NAG	2.67	Positive
MT2127	11	F	50	Túquerres	MAG	4	Positive
MT5111	12	M	50	Tumaco	MAG	3.5	Positive
MT2156	12	M	50	Túquerres	NAG	2.33	Positive
MT5126	13	M	51	Tumaco	NAG	3	Positive
MT2136	13	M	52	Túquerres	NAG	2.33	Positive
MT5116	14	F	51	Tumaco	NAG	2.33	Positive
MT2129	14	F	51	Túquerres	NAG	2	Positive
MT5155	15	M	52	Tumaco	NAG	2	Positive
MT2160	15	M	52	Túquerres	MAG	3.75	Positive
MT5174	16	M	52	Tumaco	MAG-IM	4.8	Negative
MT2113	16	M	52	Túquerres	NAG	1.67	Positive
MT5107	17	F	55	Tumaco	NAG	2.67	Positive
MT2114	17	F	55	Túquerres	MAG-IM	4.6	Positive
MT5114	18	M	56	Tumaco	MAG	3.75	Positive
MT2109	18	M	57	Túquerres	MAG-IM	4.4	Positive
MT5113	19	M	59	Tumaco	NAG	2.67	Positive
MT2112	19	M	57	Túquerres	MAG	3.5	Positive
MT5131	20	F	59	Tumaco	NAG	2.67	Positive
MT2106	20	F	60	Túquerres	MAG	3.5	Positive

**NAG**, non-atrophic gastritis; **MAG**, multifocal atrophic gastritis without intestinal metaplasia; **MAG-IM**: multifocal atrophic gastritis with intestinal metaplasia. *H. pylori* status according to Steiner stain.

**Table 2:** Results of envfit analyses testing for correlation of patient characteristics with microbiota composition patterns detected in PCoA graph.

	p value of correlation with PCoA plane	
	axis 1-axis 2	axis 1-axis 3
all samples included (n = 40)		
histological diagnosis	0.14	0.29
histopathology score	0.30	0.40
patient age	0.06	0.09
patient sex	0.55	0.19
town	**0.0012**	0.24
samples with available *cag*PAI status (n = 38)		
*cag*PAI status	0.25	0.77
samples with *H. pylori* population data (n = 33)		
% AA1	0.45	0.48
% AA1 > 20%?	0.39	0.39
% AA1 > 50%?	0.28	0.30
modern *H. pylori* population	0.47	0.42

P values were based on 10,000 permutations. P values ≤ 0.05 (bold text) are considered significant.

**Table 3:** OTUs with significantly different occurrence among towns (according to Metastats analysis), with false discovery rate q. Excluding OTUs found in one sample only.

OTU name	OTU counts in sample sets		Number of samples containing OTU		q
	Tumaco	Túquerres	Tumaco	Túquerres	
*significantly more in Túquerres*					
*Leptotrichia wadei*	1	16	1	2	0.017
OTU 508 (*Veillonella* sp.)	1	18	1	1	0.007
*significantly more in Tumaco*					
OTU 566 (*Staphylococcus* sp.)	4068	0	7	0	0.007
*Neisseria flavescens*	113	0	4	0	0.007
OTU 359 (*“Porphyromonadaceae” gen.* sp.)	42	0	4	0	0.007
OTU 438 (*Flavobacterium* sp.)	11	0	4	0	0.047
OTU 549 (*TM7-genera incertae sedis*)	18	0	3	0	0.003
OTU 430 (*Rothia* sp.)	136	0	2	0	0.007
*Prevotella oris*	39	0	2	0	0.007
*Capnocytophaga gingivalis*	33	0	2	0	0.007
OTU 541 (*Actinomyces* sp.)	31	0	2	0	0.007
OTU 486 (*Haematobacter* sp.)	25	0	2	0	0.007
OTU 362 (*Flavobacteriaceae* gen. sp.)	23	0	2	0	0.007
OTU 515 (*Actinomyces* sp.)	18	0	2	0	0.003
OTU 506 (*Sphingomonadaceae* gen. sp.)	18	0	2	0	0.003
*Streptococcus oralis*	14	0	2	0	0.008
OTU 440 (*Porphyromonas* sp.)	13	0	2	0	0.015
OTU 491 (*Neisseria* sp.)	13	0	2	0	0.015
