# Supplementary material for: Different gastric microbiota compositions in two human populations with high and low gastric cancer risk in Colombia
Source: Sci Rep. 2016 Jan 5;6:18594. doi: 10.1038/srep18594 (PMC4700446; doi:10.1038/srep18594)
Supplement: Supplementary Information [file srep18594-s1.pdf]

Different gastric microbiota compositions in two human populations with high and low gastric cancer risk in Colombia

Ines Yang, Sabrina Woltemate, M. Blanca Piazuelo, Luis E. Bravo, Maria Clara Yepez, Judith Romero-Gallo, Alberto G. Delgado, Keith T. Wilson, Richard M. Peek, Jr., Pelayo Correa, Christine Josenhans, James G. Fox, Sebastian Suerbaum

Supplementary Figures

Supplementary Tables

Supplementary Methods

Supplementary Datasets are provided as a separate file.

SUPPLEMENTARY FIGURES

Supplementary Fig. S1: Rarefaction curves, obtained after subsampling to 6960 sequences per sample. a, by town. Samples originating from age- and sex-matched participants shown in the same color. b average values of towns (solid lines), with standard deviations (dashed lines).

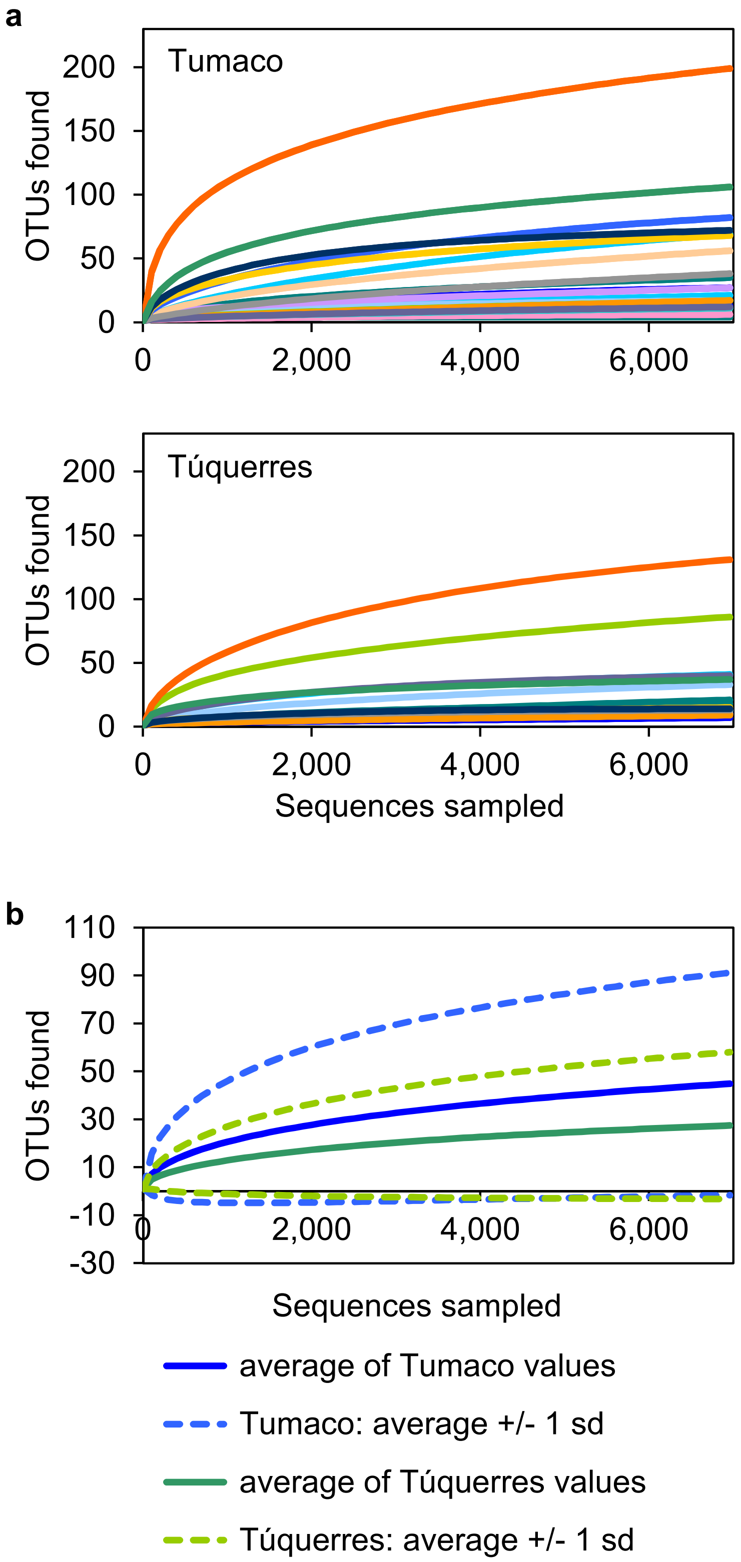

Supplementary Figure S2: Phylogeny used for calculation of UniFrac distances, with most detailed available classification added to each OTU and with local support values added to nodes. Support values below 70 not shown. Please view at high magnification.

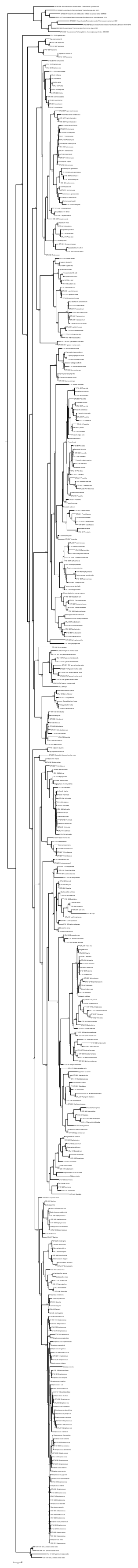

SUPPLEMENTARY TABLES

Supplementary Table S1: Sequence counts of *Helicobacteraceae* OTUs in the subsampled dataset.

OTU name	Classification (RDP classifier)	sequence counts		number of samples with OTU	
		Tumaco	Túquerres	Tumaco	Túquerres
Helicobacter pylori	*Helicobacteraceae, Helicobacter*	110421	113125	20	20
Helicobacter suis	*Helicobacteraceae, Helicobacter*	1	0	1	0
OTU_154	*Helicobacteraceae*	1	0	1	0
OTU_408	*Helicobacteraceae, Helicobacter*	1	0	1	0
OTU_496	*Helicobacteraceae, Helicobacter*	2	0	2	0
OTU_512	*Helicobacteraceae, Helicobacter*	1	1	1	1
OTU_535	*Helicobacteraceae, Helicobacter*	1	1	1	1
OTU_543	*Helicobacteraceae, Helicobacter*	1	0	1	0
OTU_563	*Helicobacteraceae, Helicobacter*	9074	10285	19	20

Supplementary Table S2: Population membership and proportion of Ancestral Africa 1 ancestry of *H. pylori* clones isolated from biopsy material, plus cagPAI status based on both biopsy material and isolated strains.

Sample ID	Modern population	Proportion of AA1 ancestry	*cag*PAI status
Tumaco			
MT5101	hpEurope	3.9%	*cag*PAI+
MT5105	hpAfrica1	61.3%	*cag*PAI+
MT5106	hpEurope	3.7%	*cag*PAI-
MT5107	hpEurope	10.1%	*cag*PAI+
MT5111	hpAfrica1	71.0%	*cag*PAI+
MT5113	hpEurope	31.2%	*cag*PAI+
MT5114	hpAfrica1	70.8%	*cag*PAI+
MT5116	hpEurope	3.4%	*cag*PAI-
MT5117	hpAfrica1	33.8%	*cag*PAI+
MT5119	n.d.	n.d.	*cag*PAI+
MT5120	hpEurope	1.6%	*cag*PAI+
MT5124	hpAfrica1	52.1%	*cag*PAI+
MT5126	hpEurope	32.3%	*cag*PAI+
MT5131	hpAfrica1	52.3%	*cag*PAI+
MT5135	hpAfrica1	60.2%	*cag*PAI+
MT5136	hpAfrica1	61.6%	*cag*PAI+
MT5139	hpAfrica1	67.7%	*cag*PAI+
MT5155	hpEurope	21.8%	*cag*PAI+
MT5174	n.d.	n.d.	n.d.
MT5176	hpEurope	17.3%	*cag*PAI+
Túquerres			
MT2102	hpEurope	9.2%	*cag*PAI+
MT2106	n.d.	n.d.	*cag*PAI+
MT2108	hpEurope	10.1%	*cag*PAI+
MT2109	n.d.	n.d.	*cag*PAI+
MT2112	hpEurope	22.9%	*cag*PAI+

MT2113	n.d.	n.d.	n.d.
MT2114	n.d.	n.d.	*cag*PAI+
MT2115	hpEurope	15.7%	*cag*PAI+
MT2118	hpEurope	22.6%	*cag*PAI+
MT2120	hpAfrica1	32.9%	*cag*PAI+
MT2122	hpEurope	13.2%	*cag*PAI+
MT2124	hpEurope	24.1%	*cag*PAI+
MT2127	hpEurope	13.0%	*cag*PAI+
MT2129	hpEurope	15.3%	*cag*PAI+
MT2130	hpEurope	3.5%	*cag*PAI+
MT2131	hpEurope	11.8%	*cag*PAI+
MT2133	hpEurope	12.7%	*cag*PAI-
MT2136	hpEurope	22.5%	*cag*PAI+
MT2156	hpEurope	9.5%	*cag*PAI+
MT2160	n.d.	n.d.	*cag*PAI+

Supplementary Table S3: Contribution of ancestral *H. pylori* populations to strains isolated from biopsies.

Sample ID	AE1	AE2	Ancestral Africa 1	Ancestral Sahul	Ancestral East Asia	Ancestral Africa 2
**Tumaco**						
MT5101	35.9%	54.7%	3.9%	0.7%	2.6%	2.2%
MT5105	7.6%	28.8%	61.3%	0.6%	0.9%	0.6%
MT5106	36.4%	54.3%	3.7%	0.6%	2.7%	2.2%
MT5107	31.2%	51.0%	10.1%	0.7%	6.7%	0.3%
MT5111	4.9%	22.2%	71.0%	0.5%	1.1%	0.3%
MT5113	25.4%	39.6%	31.2%	1.0%	2.5%	0.3%
MT5114	5.0%	22.2%	70.8%	0.6%	1.1%	0.2%
MT5116	39.4%	53.2%	3.4%	0.6%	1.2%	2.1%
MT5117	28.4%	34.3%	33.8%	1.1%	2.3%	0.2%
MT5120	37.0%	54.6%	1.6%	0.5%	5.7%	0.6%
MT5124	22.4%	18.8%	52.1%	0.6%	5.8%	0.3%
MT5126	17.5%	31.6%	32.3%	4.8%	13.4%	0.4%
MT5131	12.6%	33.7%	52.3%	0.4%	0.5%	0.4%
MT5135	0.8%	27.7%	60.2%	1.2%	9.9%	0.3%
MT5136	7.1%	28.4%	61.6%	0.8%	1.7%	0.3%
MT5139	11.7%	17.9%	67.7%	0.5%	1.9%	0.3%
MT5155	26.3%	49.7%	21.8%	0.4%	1.2%	0.4%
MT5176	32.8%	37.1%	17.3%	2.7%	6.6%	3.6%
**Túquerres**						
MT2102	25.7%	63.7%	9.2%	0.5%	0.7%	0.2%
MT2108	30.5%	51.8%	10.1%	1.4%	5.9%	0.3%
MT2112	31.6%	32.6%	22.9%	0.8%	10.2%	2.0%
MT2115	24.0%	37.5%	15.7%	0.4%	20.0%	2.3%
MT2118	27.7%	37.2%	22.6%	11.3%	1.0%	0.2%
MT2120	20.5%	41.3%	32.9%	3.3%	1.6%	0.3%
MT2122	40.7%	42.6%	13.2%	1.5%	1.8%	0.2%

MT2124	24.5%	49.9%	24.1%	0.3%	0.9%	0.3%
MT2127	32.2%	53.3%	13.0%	0.4%	0.8%	0.3%
MT2129	18.3%	64.8%	15.3%	0.4%	0.8%	0.5%
MT2130	21.4%	73.3%	3.5%	0.6%	1.0%	0.3%
MT2131	26.3%	60.7%	11.8%	0.4%	0.6%	0.3%
MT2133	26.5%	57.2%	12.7%	1.9%	1.4%	0.3%
MT2136	24.0%	42.2%	22.5%	0.4%	8.8%	2.1%
MT2156	15.5%	71.3%	9.5%	0.8%	2.7%	0.3%

Supplementary Table S4: Archaeal 16S rDNA sequences used as outgroup during phylogenetic reconstruction.

Accession	Phylum	Order	Organism name
CP001140	*Crenarchaeota*	*Desulfurococcales*	*Desulfurococcus kamchatkensis* 1221n
CP000077	*Crenarchaeota*	*Sulfolobales*	*Sulfolobus acidocaldarius* DSM 639
CP000505	*Crenarchaeota*	*Thermoproteales*	*Thermofilum pendens* Hrk 5
CP001857	*Euryarchaeota*	*Archaeoglobales*	*Archaeoglobus profundus* DSM 5631
CP001687	*Euryarchaeota*	*Halobacteriales*	*Halorhabdus utahensis* DSM 12940
HE613800	*Euryarchaeota*	*Thermococcales*	*Pyrococcus abyssi* GE5
BA000011	*Euryarchaeota*	*Thermoplasmatales*	*Thermoplasma volcanium* GSS1
DQ397549	*Thaumarchaeota*	*Cenarchaeales*	*Cenarchaeum symbiosum* A

SUPPLEMENTARY METHODS

Study participants, samples and histopathology

Subjects between 40 and 60 years old with dyspeptic symptoms that warranted upper gastrointestinal tract endoscopy were recruited in Tumaco and Túquerres in 2010. Subjects that had received proton pump inhibitors, H2-receptor antagonists, or antimicrobials during the 30 day period previous to the endoscopic procedure were excluded from this study. Other exclusion criteria were major diseases or previous gastrectomy. Participation was voluntary and informed consent was obtained from all participants. The Ethics Committees of the participating hospitals in Nariño and the Universidad del Valle in Cali, Colombia and the Institutional Review Board of Vanderbilt University approved all study protocols.

All endoscopies were performed by a single experienced gastroenterologist. Four gastric biopsy samples were obtained from all participants for histopathology: two from the antrum, one from the incisura angularis, and one from the corpus. One additional antral biopsy from each participant was frozen in glycerol/thioglycolate and stored at −80°C.

Histopathology was evaluated by two GI pathologists blinded to all demographic information. The most advanced lesion observed in the set of biopsies from each subject was considered the diagnosis. Diagnostic categories included the steps of the precancerous cascade: non-atrophic gastritis (NAG), multifocal atrophic gastritis without intestinal metaplasia (MAG), multifocal atrophic gastritis with intestinal metaplasia (MAG-IM), and dysplasia. In addition, a detailed histopathology scoring system was applied that takes into account differences in severity and extension of the lesions within each diagnostic category, as previously described[1]. Presence of *H. pylori* was assessed by histology in the entire set of biopsies for all subjects using a modified Steiner silver stain.

For the current study, 40 subjects (20 per town) were selected from a total of 182 enrolled participants. Subjects from both towns were matched by age and sex, purposely selecting those with the least advanced histological lesions possible (NAG or MAG). However, a few subjects with MAG-IM were included as there were not enough cases with less advanced lesions to complete the set of 20 pairs.

One antral frozen biopsy from each subject was shipped in dry ice to Massachusetts Institute of Technology in Cambridge, MA for extraction of DNA for *H. pylori* characterization and microbiome study.

DNA techniques

All standard procedures (DNA amplification, purification and manipulation) were performed according to standard protocols[2]. Purification of chromosomal DNA from *H. pylori* was performed using using a "High Pure PCR Template Preparation Kit" from Roche (http://www.roche-applied-science.com/shop/products/high-pure-pcr-template-preparation-kit). Where not mentioned otherwise, sequence data were analyzed and alignments were generated using BioNumerics v7.1 (Applied Maths NV).

Biopsy samples and individual *H. pylori* strains from each patient were tested for the presence of the *cag* pathogenicity island (*cag*PAI) using PCRs targeting *cagL* (HP0539), *cagN* (HP0538) and *cagA*. Broad range PCR primers were designed for each *cag* gene according to our previous data[3] such that *cag*PAI genes from all different phylogenetic groups were reproducibly amplified. PCR to detect the presence of an „empty" insertion site of the *cag* pathogenicity island (empty site PCR) was performed as described[3].

***H. pylori* multilocus haplotype analysis**

H. pylori multilocus sequence analysis based on seven housekeeping genes was performed as described[4,5]. For an assignment of strains to phylogeographic populations, the global reference haplotype set used in Nell *et al.* was modified to contain seven extra hpAsia2 haplotypes[6,7]. The reference haplotype set for investigation of hpAfrica1 subpopulations was composed of both reference and Cameroonian hpAfrica1 haplotypes mentioned in Nell *et al.*[5] Alignments were processed for STRUCTURE import using xmfa2struct[8]. Assignment of populations and subpopulations using the No Admixture Model was based on 10 independent STRUCTURE runs for different numbers of bacterial populations; analysis of ancestral population contributions was based on 15 independent STRUCTURE runs of the Linkage Model. Ancestral contributions were calculated as the average of the 5 runs with the highest probability.

Microbiota analysis

Partial amplification of 16S rDNA and deep sequencing of amplicons. The microbiota composition was analyzed as described in Yang *et al.*[9], with slight modifications. Briefly, genomic DNA was extracted from the biopsies using a modified QIAamp protocol for tissue samples (QIAGEN). 16S rRNA gene fragments were amplified using barcoded 454 Lib-L Fusion primers containing the template-specific parts 8F (5'-AGAGTTTGATCCTGGCTCAG-3') and 541R (5'-WTTACCGCGGCTGCTGG-3')[10]. Each 50 µl PCR reaction mix included 5.2 µl 10 x buffer containing 18 mM $MgCl_2$ (FastStart High Fidelity PCR System, Roche), 10 nmol of each dNTP (dNTP Set 100 mM Solutions, Life Technologies), 20 pmol of each primer, 2.5 U High Fidelity Taq polymerase (FastStart High Fidelity PCR System, Roche) and 20 ng DNA. The PCR program consisted of an initial denaturation and polymerase activation step at 94°C for 3 min, 35 cycles of 15 s at 94°C, 45 s at 60°C, and 1 min at 72°C, and a final elongation step of 8 min at 72°C. PCRs included positive and no-template negative controls. Amplification products were separated on 1% agarose gels and examined visually after ethidium bromide staining. Fragments of 550 to 650 bp length were cut out and extracted with the QIAquick Gel Extraction Kit (QIAGEN). DNA concentrations were determined using the Quant-iT PicoGreen dsDNA Kit (Life Technologies) on a Victor^3 1420 Multilabel Counter (PerkinElmer). Four to eight differently barcoded samples were pooled at 25 ng per sample. Emulsion PCR was performed following the emPCR Lib-L protocol modified according to the Long Fragment Protocol TCB11001 (both according to Roche). Samples were sequenced from the 3' end using GS Titanium chemistry on a 454 GS FLX+ instrument. Raw sequencing data were extracted with the shotgun pipeline of GS Run Processing Software version 2.6.

Bioinformatic analysis. Sequences were filtered, quality-trimmed and sorted by barcode using the mothur software version 1.25.1[11]. Sequences with more than one mismatch in the barcode or more than two mismatches in the primer region were discarded. Raw sequences were trimmed at the 3' end to remove ambiguous bases, homopolymer regions longer than 8 bp, and regions in which the average quality score over a 50 bp window was lower than 35. Sequences with a remaining length of less than 100 bp were removed from the dataset. Sequences were dereplicated and aligned using the mothur align.seqs command with the ''SILVA'' reference

alignment[12] and standard settings. The aligned dataset was preclustered at a 1 bp difference level to remove potential sequencing errors, taking into account the abundance information obtained during the dereplication step. PCR chimeras were identified and removed using UCHIME[13] via the mothur chimera.uchime command and the ‘‘SILVA gold’’ reference set[12]. Following classification with RDP Classifier version 2.5[14]. which had been slightly modified to use 1000 bootstrap replicates, chloroplast sequences and sequences that could not be identified to class were excluded from the final dataset. Sequences identified as *Alphaproteobacteria* were tested by blastn search using BLAST version 2.2.26+[15] against the SILVA reference database, build 108[16] to check for mitochondrial sequences. For species identification, we used a modified version of RDP 16S rRNA database release 10.29 that had been processed with TaxCollector 2.0[17] for easier automatic extraction of taxonomic classifications. In order to avoid memory constraints, the query dataset was split into subsets of 10 sequences each, which were searched against the TaxCollector-modified RDP database; blastn searches were capped at 50,000 hit sequences, 97% minimum identity and a minimal BLAST e-value setting of 1E-50. Sequences were considered as classified to species if they had been identified to genus at a bootstrap value over 0.97 during RDP classifier analysis, their identity to the best hit in the TaxCollector-modified database was at least 97%, the aligned region covered at least 97% of the query sequence length, they did not match a second species at the same BLAST expectation value, and the species classification of the best hit did not conflict with the genus classification obtained using the RDP classifier. Sequences that could not be classified to species were clustered into operative taxonomic units (OTUs) using ESPRIT-Tree[18]. The individual reads within each OTU were aligned using MUSCLE[19], or MAFFT[20] with the settings “FFT-NS-2–ep 0.123” if the OTU contained too many sequences for MUSCLE to run successfully. Based on these alignments, majority-rule consensus sequences were calculated within Geneious Basic v. 5.6.4[21]. For OTU classification, consensus sequences were analysed with the RDP classifier as detailed above. Additionally, 80% consensus classifications of the individual sequences were calculated for each OTU. The more detailed of these two classifications was assigned to the OTU (Supplementary Dataset S8). Identified species and OTUs were combined into one dataset. The dataset was subsampled to 6960 sequences per sample so as to match the sample with the lowest number of sequences. This subsampled dataset, as well as rarefaction curves, a Venn diagram, Jaccard distance matrices and Analysis of Molecular Variance (AMOVA) statistics were calculated using mothur. The Venn diagram was displayed using the Venn Diagram Generator and edited for better readability and additional text

using Inkscape 0.48.

Overall patterns of microbiota similarity between samples were examined using Principal Coordinates Analysis (PCoA) based on UniFrac distances. These UniFrac distances were based on a rooted phylogenetic tree of OTU consensus sequences, which was constructed as follows: OTU consensus sequences were aligned to the 8F-541R region of the bacterial sequences within the SILVA_119_SSURef_Nr99 reference alignment[22] using the NAST algorithm[23] as implemented in usearch8.0.1517[24]. An outgroup of eight sequences selected to represent a broad selection of the phylogenetic diversity of *Archaea*[25] (Supplementary Table S4) was extracted from the complete SILVA_119_SSURef_Nr99 alignment, and the alignment positions corresponding to the bacterial 8F-541R amplicons (positions 1044-13125) were added to the aligned OTU consensus sequences. Gaps common in all sequences were removed using mothur v.1.33.2. A phylogenetic tree was calculated using FastTree[26] with the GTR + CAT model of nucleotide evolution and the –gamma option. The tree was rooted and the outgroup was removed using SeaView version 4[27]. An unweighted UniFrac distance matrix was calculated within the R package phyloseq[28]. Based on these distances, PCoAs were calculated with the R package vegan[29]. In order to investigate correlations with the available patient and sample characteristics, non-microbiota data were fitted to the first two planes of this ordination using the vegan function envfit. OTUs and species that occurred in more than one sample of the subsampled dataset were tested for differences of abundance in Tumaco or Túquerres using Metastats[30] as implemented in the original R script.

In order to further test for possible associations between patient characteristics and individual OTUs, we used correlation analysis based on Spearman's rank correlation, or in the case of categorical data categorical factor regression, with Benjamini-Hochberg-correction (false discovery rate ≤ 0.05) as implemented in R 3.0.2. The calculations were based on three different versions of the OTU dataset: Relative abundances of the non-*Helicobacteraceae* OTUs in the subsampled dataset, a binary version of the subsampled dataset containing presence-absence-data of non-*Helicobacteraceae* OTUs, and relative abundances of the non-*Helicobacteraceae* OTUs in the non-subsampled dataset. From each of these datasets, OTUs present in less than 2 samples were removed prior to analysis.

SUPPLEMENTARY METHODS REFERENCES

1. de Sablet, T. *et al.* Phylogeographic origin of *Helicobacter pylori* is a determinant of gastric cancer risk. *Gut* **60,** 1189–1195 (2011).
2. Sambrook, J. & Russell, D. G. *Molecular cloning: a laboratory manual.* **3,** (Cold Spring Harbor Laboratory Press, 2004).
3. Olbermann, P. *et al.* A global overview of the genetic and functional diversity in the *Helicobacter pylori cag* pathogenicity island. *PLoS Genet.* **6,** e1001069 (2010).
4. Falush, D. *et al.* Traces of human migrations in *Helicobacter pylori* populations. *Science* **299,** 1582–1585 (2003).
5. Nell, S. *et al.* Recent acquisition of *Helicobacter pylori* by Baka pygmies. *PLoS Genet.* **9,** e1003775 (2013).
6. Didelot, X. *et al.* Genomic evolution and transmission of *Helicobacter pylori* in two South African families. *Proc. Natl. Acad. Sci. U. S. A.* **110,** 13880–13885 (2013).
7. Wattam, A. R. *et al.* PATRIC, the bacterial bioinformatics database and analysis resource. *Nucleic Acids Res.* **42,** D581–591 (2014).
8. Didelot, X. *xmfa2struct*. at <www.stats.ox.ac.uk/~didelot/files/xmfa2struct.zip>
9. Yang, I. *et al.* Intestinal microbiota composition of interleukin-10 deficient C57BL/6J mice and susceptibility to *Helicobacter hepaticus*-induced colitis. *PLoS ONE* **8,** e70783 (2013).
10. Lofgren, J. L. *et al.* Lack of commensal flora in *Helicobacter pylori*–infected INS-GAS mice reduces gastritis and delays intraepithelial neoplasia. *Gastroenterology* **140,** 210–220 (2011).
11. Schloss, P. D. *et al.* Introducing mothur: open-source, platform-independent, community-supported software for describing and comparing microbial communities. *Appl. Environ. Microbiol.* **75,** 7537–7541 (2009).
12. Schloss, P. D. Silva reference files - mothur. (2011). at <http://www.mothur.org/wiki/Silva_reference_files>
13. Edgar, R. C., Haas, B. J., Clemente, J. C., Quince, C. & Knight, R. UCHIME improves sensitivity and speed of chimera detection. *Bioinforma. Oxf. Engl.* **27,** 2194–2200 (2011).
14. Wang, Q., Garrity, G. M., Tiedje, J. M. & Cole, J. R. Naïve Bayesian classifier for rapid assignment of rRNA sequences into the new bacterial taxonomy. *Appl. Environ. Microbiol.* **73,** 5261–5267 (2007).

15. Zhang, Z., Schwartz, S., Wagner, L. & Miller, W. A greedy algorithm for aligning DNA sequences. *J. Comput. Biol. J. Comput. Mol. Cell Biol.* **7,** 203–214 (2000).
16. Pruesse, E. *et al.* SILVA: a comprehensive online resource for quality checked and aligned ribosomal RNA sequence data compatible with ARB. *Nucleic Acids Res.* **35,** 7188–7196 (2007).
17. Giongo, A., Davis-Richardson, A. G., Crabb, D. B. & Triplett, E. W. TaxCollector: Modifying current 16S rRNA databases for the rapid classification at six taxonomic levels. *Diversity* **2,** 1015–1025 (2010).
18. Cai, Y. & Sun, Y. ESPRIT-Tree: hierarchical clustering analysis of millions of 16S rRNA pyrosequences in quasilinear computational time. *Nucleic Acids Res.* **39,** e95 (2011).
19. Edgar, R. C. MUSCLE: multiple sequence alignment with high accuracy and high throughput. *Nucleic Acids Res.* **32,** 1792–1797 (2004).
20. Katoh, K., Misawa, K., Kuma, K. & Miyata, T. MAFFT: a novel method for rapid multiple sequence alignment based on fast Fourier transform. *Nucleic Acids Res.* **30,** 3059–3066 (2002).
21. Drummond, A. *et al. Geneious*. (2012). at <http://www.geneious.com>
22. Quast, C. *et al.* The SILVA ribosomal RNA gene database project: improved data processing and web-based tools. *Nucleic Acids Res.* **41,** D590–596 (2013).
23. DeSantis, T. Z. *et al.* NAST: a multiple sequence alignment server for comparative analysis of 16S rRNA genes. *Nucleic Acids Res.* **34,** W394–399 (2006).
24. Edgar, R. C. Search and clustering orders of magnitude faster than BLAST. *Bioinforma. Oxf. Engl.* **26,** 2460–2461 (2010).
25. Petitjean, C., Deschamps, P., López-García, P. & Moreira, D. Rooting the domain Archaea by phylogenomic analysis supports the foundation of the new kingdom Proteoarchaeota. *Genome Biol. Evol.* **7,** 191–204 (2014).
26. Price, M. N., Dehal, P. S. & Arkin, A. P. FastTree 2--approximately maximum-likelihood trees for large alignments. *PloS One* **5,** e9490 (2010).
27. Gouy, M., Guindon, S. & Gascuel, O. SeaView version 4: A multiplatform graphical user interface for sequence alignment and phylogenetic tree building. *Mol. Biol. Evol.* **27,** 221–224 (2010).
28. McMurdie, P. J. & Holmes, S. phyloseq: an R package for reproducible interactive analysis and graphics of microbiome census data. *PloS One* **8,** e61217 (2013).

29. Oksanen, J. *et al. vegan: Community Ecology Package*. (2013). at <http://CRAN.R-project.org/package=vegan>
30. White, J. R., Nagarajan, N. & Pop, M. Statistical methods for detecting differentially abundant features in clinical metagenomic samples. *PLoS Comput. Biol.* **5,** e1000352 (2009).
